# Electrospinning Processing Techniques for the Manufacturing of Composite Dielectric Elastomer Fibers

**DOI:** 10.3390/ma14216288

**Published:** 2021-10-22

**Authors:** Mirella Ramirez, Louis Vaught, Chiu Law, Jacob L. Meyer, Rani Elhajjar

**Affiliations:** 1 Department of Civil & Environmental Engineering, Department of Electrical Engineering, College of Engineering & Applied Science, University of Wisconsin-Milwaukee, 3200 N Cramer St., Milwaukee, WI 53211, USA; ramirez.mirella@gmail.com (M.R.); lawc@uwm.edu (C.L.); 2 ATSP Innovations, 6762 Shadyvilla Ln Bldg #3, Houston, TX 77055, USA; louis.vaught@atspinnovations.com (L.V.); jacob.meyer@atspinnovations.com (J.L.M.)

**Keywords:** composites, electrospinning, dielectric elastomers, fibers, actuators

## Abstract

Dielectric elastomers (DE) are novel composite architectures capable of large actuation strains and the ability to be formed into a variety of actuator configurations. However, the high voltage requirement of DE actuators limits their applications for a variety of applications. Fiber actuators composed of DE fibers are particularly attractive as they can be formed into artificial muscle architectures. The interest in manufacturing micro or nanoscale DE fibers is increasing due to the possible applications in tissue engineering, filtration, drug delivery, catalysis, protective textiles, and sensors. Drawing, self-assembly, template-direct synthesis, and electrospinning processing have been explored to manufacture these fibers. Electrospinning has been proposed because of its ability to produce sub-mm diameter size fibers. In this paper, we investigate the impact of electrospinning parameters on the production of composite dielectric elastomer fibers. In an electrospinning setup, an electrostatic field is applied to a viscous polymer solution at an electrode’s tip. The polymer composite with carbon black and carbon nanotubes is expelled and accelerated towards a collector. Factors that are considered in this study include polymer concentration, solution viscosity, flow rate, electric field intensity, and the distance to the collector.

## 1. Introduction

Dielectric elastomers (DE) are novel composite architectures capable of large actuation strains and the ability to be formed into a variety of actuator configurations. However, the high voltage requirement of DE actuators limits their applications for a variety of applications. Fiber actuators composed of DE fibers are particularly attractive as they can be formed into artificial muscle architectures to realize high actuation forces and potentially large displacements [1]. DE fibers have several important advantages with respect to potential device concepts using artificial muscles. These include rapid response time (on the scale of millisecond), the ability to hold strains under DC activation, can induce relatively large actuation forces, have high mechanical energy densities, and that they can be operated at room temperature for large numbers of cycles [2,3]. The interest in manufacturing micro or nanoscale composite fibers is increasing due to the possible applications in tissue engineering, filtration, drug delivery, catalysis, protective textiles, and sensors [4,5,6,7,8,9,10,11,12,13]. Drawing, self-assembly, template-direct synthesis, and electrospinning processing have been explored to manufacture these fibers. Electrospinning has been proposed because of its ability to produce sub-mm diameter size fibers [14,15,16]. The electrospinning technique was previously used to produce nanofibers with a diameter less than 40 nm [17], and uniaxially aligned nanofibers were fabricated with a variation of the electrospinning process [18].

Numerous studies have been made focusing on the mathematically modeling of the electrospinning process with the goal of getting in-depth insights on the factors that control the electrospinning process [19,20,21,22]. A boundary element approach for simulation of thin, charged jets was proposed for computation of electrostatic interactions of the jet and electrodes. The short-range and long-range electrostatic forces were evaluated and compared with experimental results [23]. A discrete finite difference bead model has been proposed to model the bending instability in a polymeric viscoelastic jet [24]. Previously a mathematical approach was used to model the secondary electrostatic field created by a finite length hollow cylinder in the electrospinning process [25]. Artificial neural network and response surface methodology have also recently been proposed based on the Box-Behnken design to produce the fiber with the minimum diameter. In this approach, multilayer perceptron neural networks are trained to predict the polycaprolactone fiber diameter [26]. Recent approaches have also looked to include the effect of air drag and gravity forces [27], and solvent evaporation [28,29].

**Figure 1:**
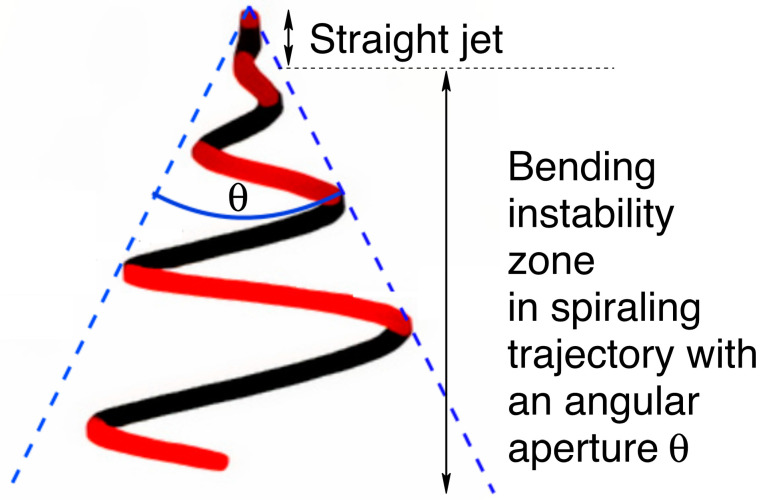
Electrospinning jet trajectory.

In this paper, we investigate using theoretical and experimental aspects the parameters that can be modulated during the production of dielectric elastomer fibers using the electrospinning process. In an electrospinning setup, an electrostatic field is applied to a viscous polymer solution at an electrode’s tip. The polymer is expelled and accelerated towards a collector. Figure 1 shows an illustration of a typical electrospinning jet trajectory. Factors that are considered in this study include polymer concentration, solution viscosity, flow rate, electric field intensity, and the distance to the collector.

## 2. Materials and Methods

**Figure 2:**
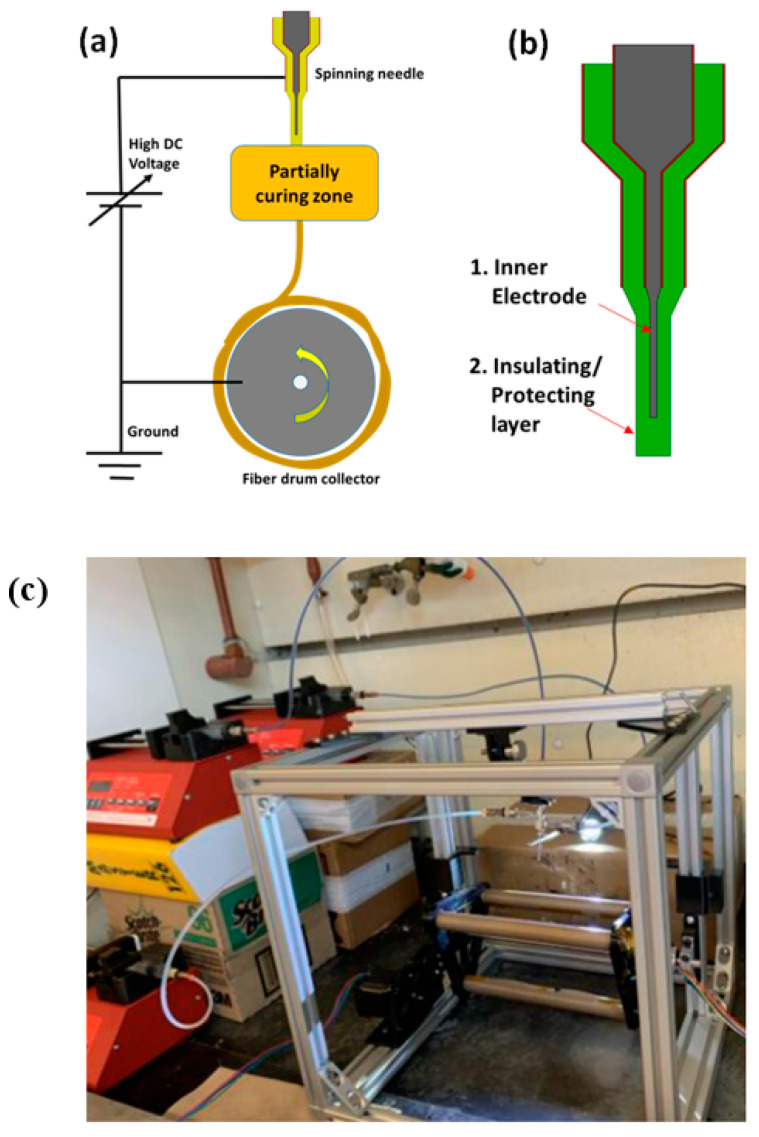
(**a**) Schematic of electrospinning setup with the drum operating at speeds of 55–80 rpm, (**b**) the nozzle, and (**c**) the assembled electrospinning setup.

**Figure 3:**
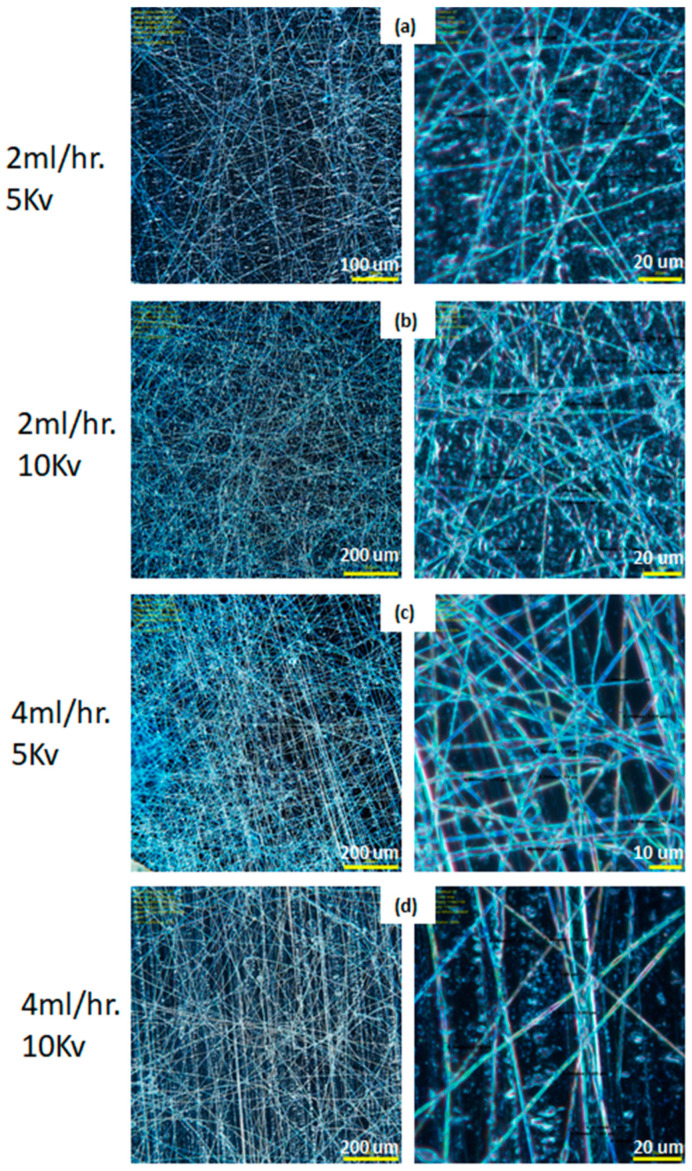
Micro image of electrospun single layer of fiber: A85 elastomer +10% carbon black illuminated by UV light, with different magnification, fiber diameter ranges from 1.3–2.9 μm at the following processing parameters (**a**) 2 mL/h at 5 kV, (**b**) 2 mL/h at 10 kV, (**c**) 4 mL/h at 5 kV, and (**d**) 4 mL/h at 10 kV.

Three polymers, A85 (Huntsman, The Woodlands, TX, USA, Irogran A85-4350 Thermoplastic polyurethane), SBS (Sigma-Aldrich, St. Louis, MO, USA, Polystyrene/polybutadiene, 30% polystyrene), SIS-17 (Sigma-Aldrich, St. Louis, MO, USA, Polystyrene/polyisoprene, 17% polystyrene), in addition of two solvents, DMF (Alfa Aesar, Haverhill, MA, USA, A13547 N, N-Dimethylformamide, 99%) and DCE (Alfa Aesar, Haverhill, MA, USA, A12775 1,2-Dichloroethane, 99+%) were examined in this study. Cabot VXC72R (Cabot, Boston, MA, USA, VXC72R) was used as conductive filler. A85 dissolves in DMF while SBS and SIS-17 dissolve in DCE with negligible mutual solubility. The viscosity profile for SBS is shown in Table 1 as measured by a spindle-type viscometer (Cole-Parmer, Vernon Hills, IL, USA, 98965). A custom electrospinning setup for spinning multilayer fibers was constructed and is shown in Figure 2. The setup consists of 1–3 syringe pumps (Southpointe Surgical, Coral Springs, FL, USA, NE-300), a custom-built rotational drum, a high voltage power supply (Acopian, Easton, PA, USA, P030HP2), and coaxial syringe (rame-hart, Succasunna, NJ, USA, 100-10-TRIAXIAL). The reason this material combination was selected was because it has sufficient conductivity and a high strain to failure which is important for the application considered. Other material combinations examined had extremely high resistances. Stable parameters for electrospinning conductively filled polymers are a distance between needle and collector of 2.5 cm, pump flow rate vs. cross-section of 2–4 mL/h, spinning voltage of 5–10 kV, and linear collector feed rate of 400–600 mm/s. As seen in Figure 3, the spun fiber is very fine (around 2 μm) and often meshed/tangled together. A summary of utilized parameters is shown in Table 2.

**Figure 4:**
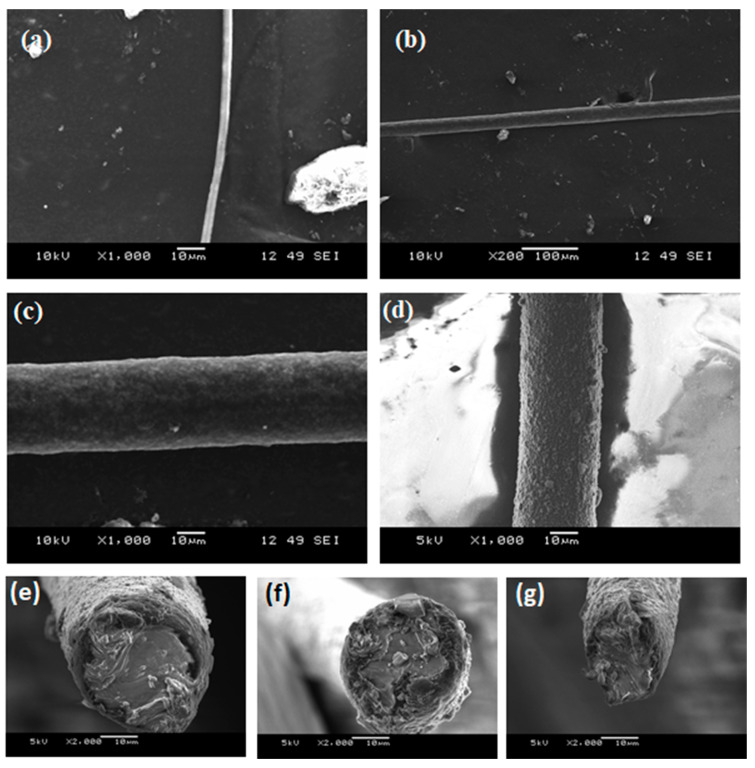
SEM images of (**a**) solution electrospun neat A85 fiber; extruded neat A85 fiber, (**b**) low magnification, and (**c**) high magnification, (**d**) 2-layer fiber with A85 core with carbon-black (**e**–**g**) examples of cross section that shows large deformation due to compression from the razor blade.

As discussed in the above electrospinning simulation section, there are few key parameters that need to be adjusted to produce the desired fiber; these include the variable speed collector drum assembly, triaxial needle, and three syringe pumps. Figure 3 shows the single layer of A85 + 10 wt.% Carbon black fiber that is electrospun by the parameters as in Table 2. For this set of experiments, concentration of solid in solvent and distance between needle and collector were fixed; the pump flow rate and spinning voltage varied to check the produced fiber diameter. Figure 4a shows the electrospun A85 fiber within 1–3 μm and as seen in Figure 3, we showed that the fiber can be tangled together and difficult to extract individual free-standing fibers following electrospinning, thus the fiber is difficult to handle with our current capabilities. For extruded A85 fibers, as shown in Figure 4b,c, the diameter is quite uniform (±2.5%) and easy to handle. As shown in Figure 4e–g of a few cross-section SEM images of two-layer fibers that have large deformation on each layer, this deformation makes the analysis of the layers more difficult. The DE fibers were tested mechanically, and we achieved 5.2 to 7.8 MPa in tension strength with a stretch ratio between 1.8 and 2.2.

## 3. Modeling of Electrospinning Process

In this study, we use an open-source simulation package JETSPIN [30] to understand the angular aperture, fiber radius, and their dependency on parameters, such as applied electric voltage, viscosity, and distance at the collector. The model implemented in JETSPIN is a discrete Lagrangian model and provides a compromise of efficiency and accuracy. Here the jet is modeled as a body constituted by a viscoelastic Maxwell fluid, and it is represented as a series of discrete elements (beads). Each ith bead has a mass $m_{i}$ and charge $q_{i}\;$Stress $\sigma_{i}$ on the ith, dumbbell which connects the ith bead with the bead i + 1 is given by the equation [30]:

(1) $$\frac{d\sigma_{i}}{dt}=\frac{G}{l_{i}}\frac{dl_{i}}{t}-\frac{G}{\mu }\sigma_{i}\;\;$$

where G is the elastic modulus, μ the viscosity of the fluid jet, and t is the time. The length $l_{i}$ has computed as the mutual distance between ith bead and its previous bead. Various forces are exerted at the ith bead, including the viscoelastic force ${\vec{f}}_{ve,i}$, the surface tension force ${\vec{f}}_{st,i}$, the electric force ${\vec{f}}_{el,i}$, the net Coulomb force ${\vec{f}}_{c,i}$, gravity force ${\vec{f}}_{g,i},$ while the air drag is modeled as a dissipative force ${\vec{f}}_{diss,i}$ and a random force ${\vec{f}}_{rand,i}$. The viscoelastic force is the force pulling bead i back to bead i − 1, and towards bead i + 1 is given by

(2) $${\vec{f}}_{ve,i}=-\pi a_{i}^{2}\sigma_{i}{\vec{t}}_{i}+\pi a_{i+1}^{2}\sigma_{i+1}{\vec{t}}_{i+1}\;.$$

where $a_{i}$ is the cross-sectional radius of the filament at the *i*-th bead and ${\vec{t}}_{i}$ is the unit vector pointing bead *i* from bead i − 1. The force acting to restore the rectilinear shape of the bending part of the jet is:

(3) $${\vec{f}}_{st,i}=\kappa_{i}\pi {\left(\frac{a_{i}+a_{i+1}}{2}\right)}^{2}\alpha {\vec{c}}_{i}$$

where α is the surface tension, $\kappa_{i}$ is the local curvature, and ${\vec{c}}_{i}$ is the unit vector pointing toward the center of the local curvature from the bead i. The force due to the electric potential between the spinneret and a conducting collector located at a distance h is ${\vec{f}}_{el,i}=\frac{e_{i}V_{0}}{h}\;\vec{z}$ and $\vec{z}$ is the unit vector pointing toward the collector from the spinneret. The net Coulomb force acting on the i-th bead from all the other beads is given by

(4) $${\vec{f}}_{c,i}=\overset{n}{\underset{j=1,\;j\neq i}{\sum }}\frac{q_{i}q_{j}}{R_{ij}^{2}}{\vec{u}}_{ij}$$

$R_{ij}={\left|{\vec{x}}_{i}-{\vec{x}}_{j}\right|}^{2}$ and ${\vec{u}}_{ij}$ is the unit vector point to the i-th bead from *j*-th bead. The force due to gravitational acceleration is ${\vec{f}}_{g,i}=m_{i}g{\vec{x}}_{i}$. The dissipative force is the sum of the longitudinal force ${\vec{f}}_{air,i}$ and the lateral force ${\vec{f}}_{lift,i}$

(5) $${\vec{f}}_{diss,i}={\vec{f}}_{air,i}+{\vec{f}}_{lift,i}$$

where ${\vec{f}}_{air,i}=-m_{i}\gamma_{i}l_{i}^{0.905}v_{t,i}^{1.19}{\vec{t}}_{i-1}$, with $\gamma_{i}=0.65\pi \frac{\rho_{a}}{m_{i}}\;{\left(\frac{2}{\nu_{a}}\right)}^{-0.81}l_{i}^{0.095}a_{i}^{0.19}$, $\rho_{a}$ the air density, $\nu_{a}$ the kinematic viscosity, $v_{t}=\left(\vec{v}-{\vec{v}}_{f}\right)\cdot \vec{t}$ representing the tangent component of the total velocity with respect to the air flow velocity ${\vec{v}}_{f}$ for a jet traveling with velocity $\vec{v}$ and ${\vec{f}}_{lift,i}=-l_{i}\kappa_{i}\rho_{a}v_{t,i}^{2}\pi {\left(\frac{a_{i}+a_{i-1}}{2}\right)}^{2}{\vec{c}}_{i}$. The random force, ${\vec{f}}_{rand,i}={\left(2m_{i}^{2}D_{v}\right)}^{1/2}\;{\vec{\eta }}_{i}\left(t\right)$, where $D_{v}\;$is a generic diffusion coefficient in velocity space, ${\vec{\eta }}_{i}\left(t\right)$ is a 3D vector obtained from the temporal derivative of ς(t) a stochastic process with stationary independents increments. The combined action of these forces governs the elongation of the jet according to Newton’s equation providing a non-linear Langevin-like stochastic differential equation:

(6) $$m_{i}\frac{d{\vec{v}}_{i}}{dt}={\vec{f}}_{ve,i}+{\vec{f}}_{st,i}+{\vec{f}}_{el,i}+{\vec{f}}_{c,i}+{\vec{f}}_{g,i}+{\vec{f}}_{diss,i}+{\vec{f}}_{rand,i}$$

where ${\vec{v}}_{i}$ is the velocity of the i-th bead and it satisfies the kinematic relation, $\frac{d{\vec{r}}_{i}}{dt}={\vec{v}}_{i}$ and ${\vec{r}}_{i}$ is the position vector of the i-th bead. This set of motion equations governs the time evolution of the system. The model also includes the fast-mechanical oscillations of the spinneret adding small perturbations to $x_{n},\;y_{n}$ coordinates of the nozzle. Given the initial position of the nozzle with the initial phase φ and the amplitude of the perturbation A

(7) $$x_{n}=A\cos\varphi ,\;y_{n}=A\sin\varphi$$

The equations of motion for the nozzle bead are

(8) $$\frac{dx_{n}}{dt}=-\omega x_{n},\;\frac{dy_{n}}{dt}=\omega y_{n}$$

where ω is the angular frequency. In this model, the simulation starts with only two bodies, a single mass-less point fixed at z=0, representing the spinneret nozzle, and a single bead modeling as a jet segment of mass $m_{i}$ and charge $e_{i}$ of length $l_{i}=l_{step}.$ The initial bead is assumed to have a cross-section radius $a_{0}$, defined as the filament radius at the nozzle before the stretching starts. In his simulation, $a_{0}$ is an input parameter, and it is directly related to the nozzle-radius. To integrate the homogeneous differential equations of motion, the time is discretized as $t_{i}=t_{0}+i\Delta t$. The software has three different schemes for the integration: Euler, Heun, and fourth-order Runge–Kutta. They note a general agreement between simulations and experimental results as reported in [31]. In [27],the dynamics of electrified polymer jets under different conditions of air drag. Here, the controlled gas counter-flow might decrease the mean value of the fiber cross-sectional radius.

## 4. Results and Discussion

**Figure 5:**
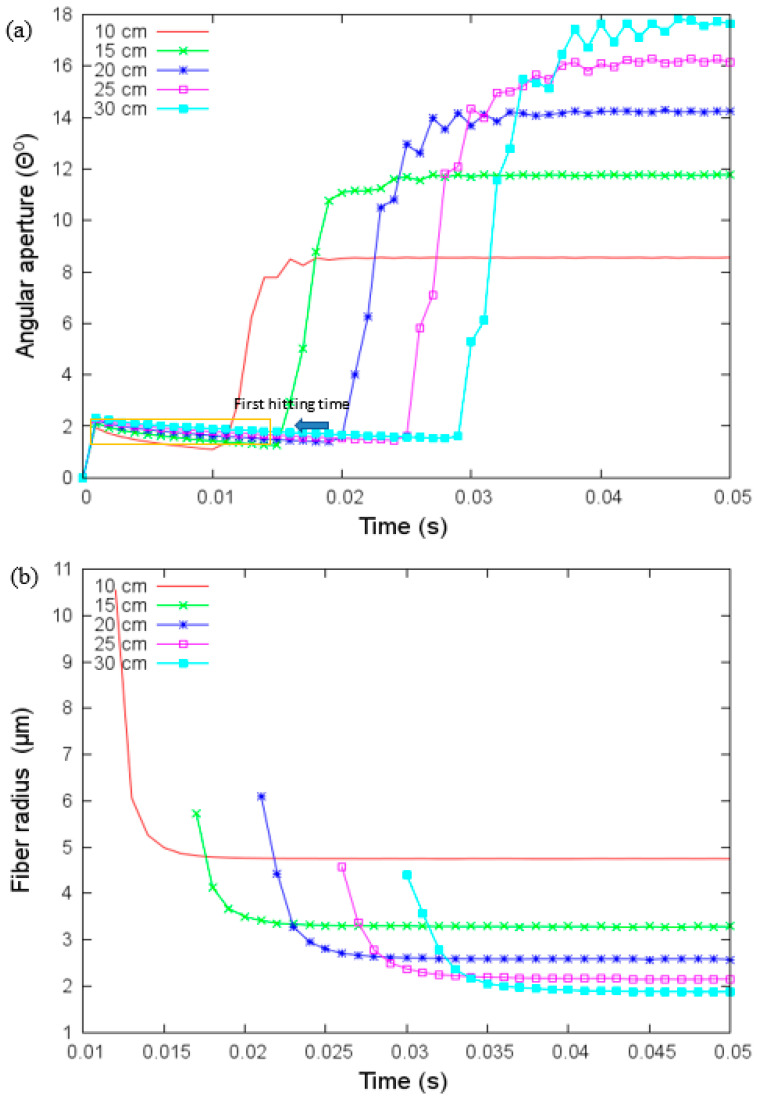
(**a**) Angular aperture versus time and (**b**) fiber radius versus time for different values of the collector’s distance under parameters given in Table 3. The first hitting time occurs when the jet touches the collector.

**Figure 6:**
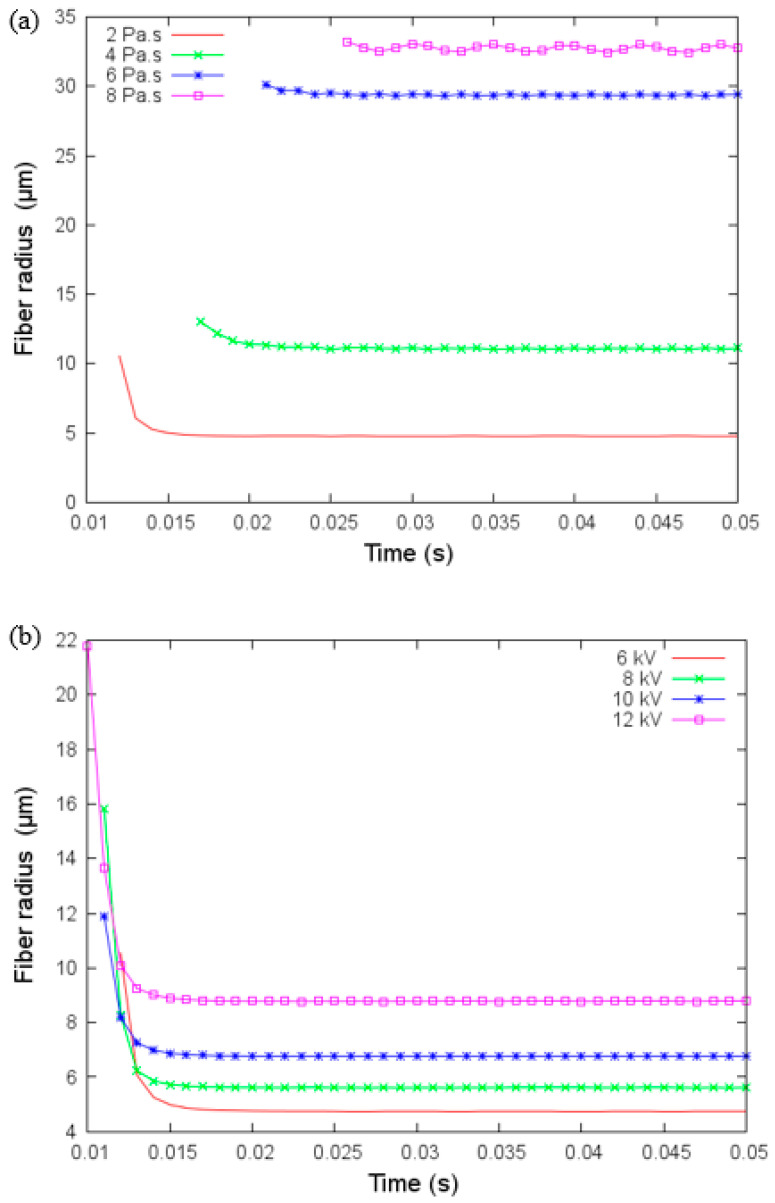
(**a**) Fiber radius versus time for different values of viscosity and (**b**) fiber radius versus time for different values of applied voltage under parameters given in Table 3.

**Figure 7:**
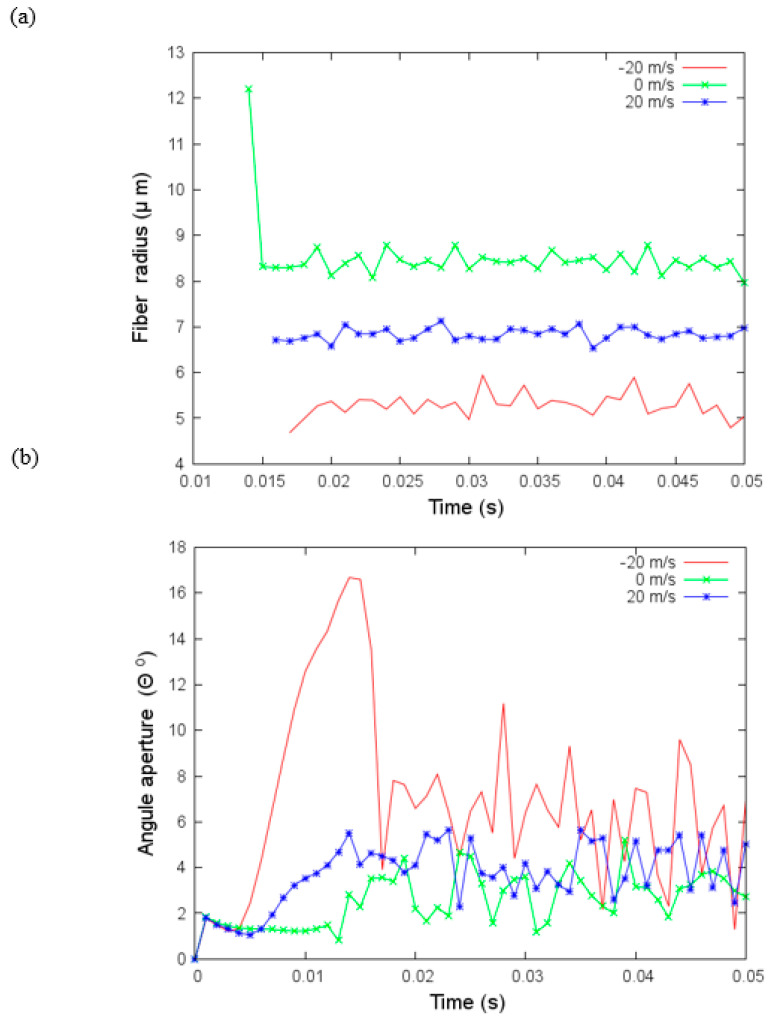
(**a**) Angular aperture versus time and (**b**) fiber radius versus time for different values of the velocity of the flow, $v_{f}$ under parameters given in Table 3.

**Figure 8:**
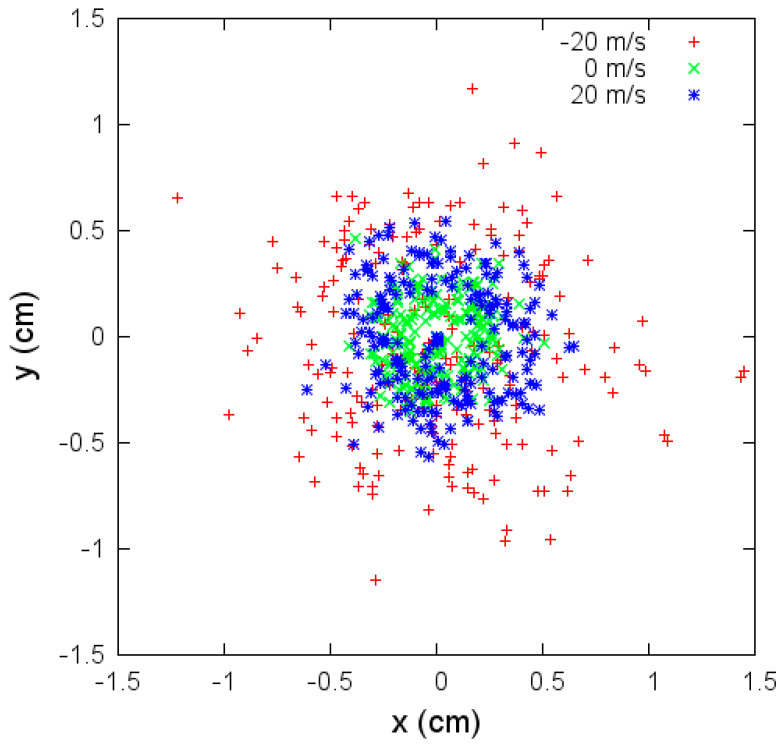
Coordinates x and y where the jet bead hits the collector for different flow velocities, $v_{f}$ under parameters given in Table 3.

**Figure 9:**
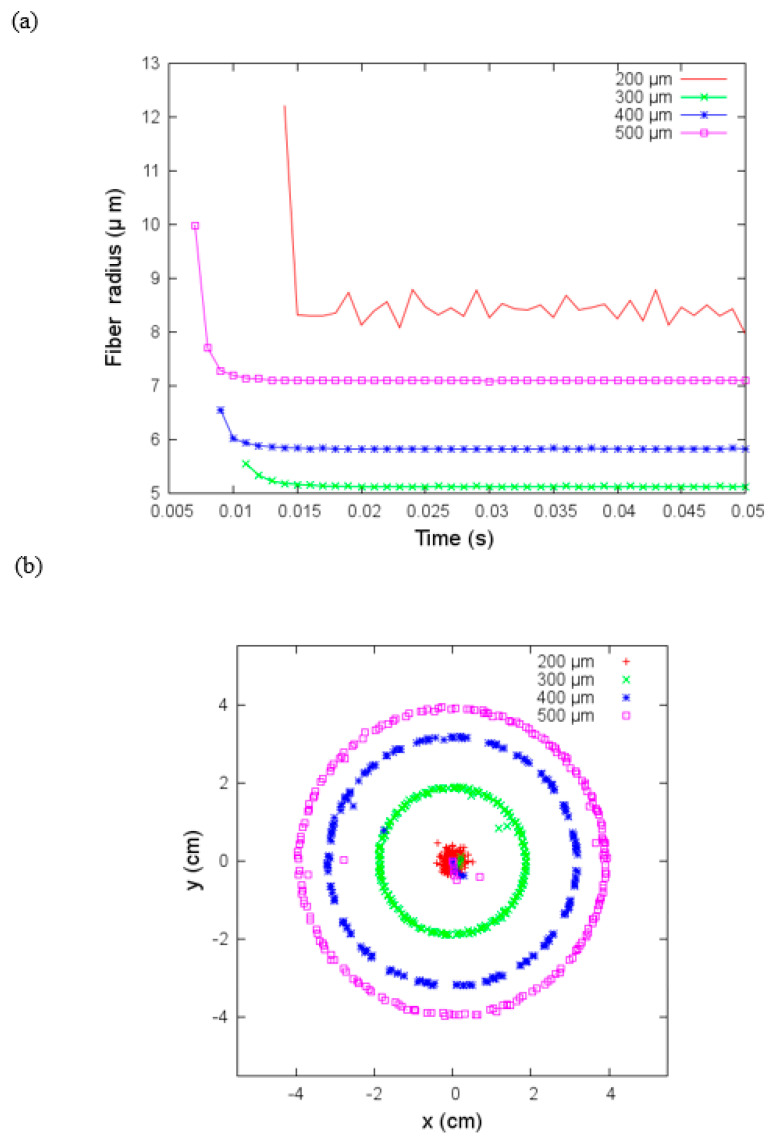
(**a**) Fiber radius versus time and (**b**) coordinates on the x-y plane where the jet hits the collector for different sizes of nozzle radius with flow velocity equal to zero under parameters given in Table 3.

**Figure 10:**
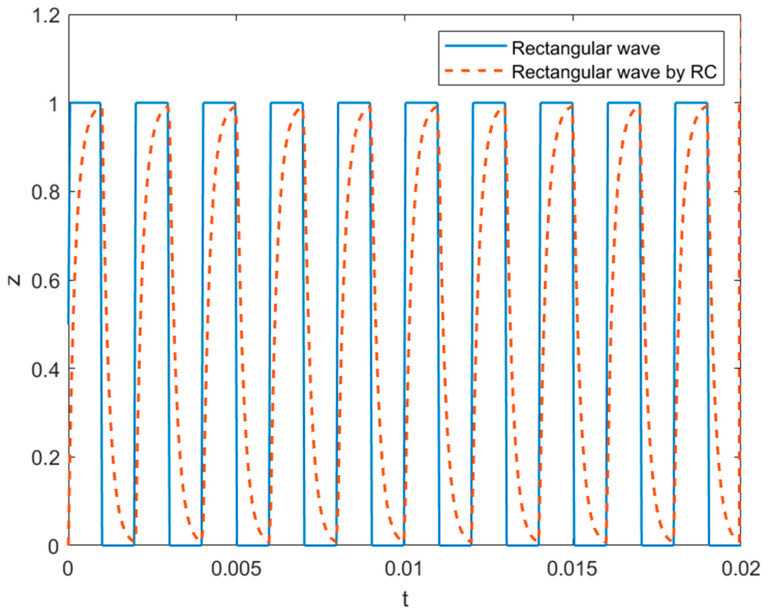
The rectangular waveform at $f_{s}=0.5\times 10^{3}\;\mathrm{Hz}\;$and its variant with the relaxation time constant RC=0.2 ms used in simulations.

The electrospinning has two stages: the uniaxial elongation of the extruder polymer jet and the second one characterized by a bending instability related to the angular aperture *θ*. *θ* increases the path traveled (see Figure 1), and with this, the fiber radius becomes smaller. We show the effect of the distance at the collector, viscosity, the applied voltage on the fiber radius, and angular aperture in the Figure 4, Figure 5, Figure 6, Figure 7, Figure 8, Figure 9 and Figure 10. We performed simulations with properties of the elastomer and modeling parameters listed in Table 3. In the simulation we are assuming that the elastomer has a controlled viscosity between 2 Pa and 20 Pa. Applied voltage, distance at the collector and nozzle radius have no such restriction and the values used were based on our test setup.

### 4.1. Effect of Collector Distance

We analyzed first the effect of distance at the collector in Figure 5. The angular aperture in Figure 5a shows three stages: at the first stage, the angle is small and increases slowly, the second stage occurs when the jet touches the collector, then the angle has a significant increase, and the third stage, the angle converges to a unique value. As the distance at the collector increases, variations in the angular aperture are present. Angular aperture increases as a function of the distance at the collector, and fiber radius decreases as a function of the distance at the collector (see Figure 5b) as it is expected. For a distance larger than 20 cm the changes in the fiber radius are small, i.e., after 20 cm, there is no significant dependence on the placement of the collector. The time to reach the collector increases as the distance increases.

### 4.2. Effect of Viscosity and Applied Voltage

Figure 6a,b show the fiber radius against time for different viscosity values and applied voltage, respectively. Fiber radius increases as a function of the viscosity and voltage applied. The baseline value for viscosity in the modeling was 2 Pa, but when we increased this to 4, 6, and 8 Pa, we show that the rate of change in fiber diameter is reduced as the viscosity is increased. Viscosity also has a more significant influence at lower levels since the change can be reaching a value greater than 30 μm in fiber radius while changing the collector distance and applied voltage enables the radius to reach almost 10 μm under fixed viscosity of 2 Pa. Since the fiber radius size varies smoothly with the applied voltage, the applied voltage could be a key parameter for the fine control of the fiber radius size.

### 4.3. Effect of Air Drag on Polymer Jet

In the next simulations (Figure 7), we added the drag effects of a gas flow with the air density and viscosity in Table 3 and three airflow velocities, $v_{f}=-20,\;0,\;20\;m/s$. We need to point out that negative velocity indicates that the gas flow is in the opposite direction of the jet and positive velocity that it is the same direction of the jet. The random force added is modeled with an amplitude of one. Fiber radius is thicker if the airflow has a velocity that is different from zero. If it is in the same direction as the jet, the reduction of the fiber radius is smaller; moreover, the fiber radius variations are also smaller. With a velocity equal to zero, the fiber radius is between 8 and 9 microns which is larger than those without the air drag effects. This difference is caused by the addition of the air density and viscosity in the current simulations.

Variations of angular aperture and fiber radius over time under aerodynamic drag effects are investigated in the next set of simulations. These changes are more significant when the velocity is negative. The variations affect the fibers’ deposition. In Figure 8, we show the coordinates *x* and *y* where the jet bead hits the collector. In the case of negative velocity flow, $v_{f}=-20\;m/s$, the deposition is quite irregular, and the area where the fibers are deposited is more extensive than in the other two cases. In the case without flow velocity ($v_{f}=0\;m/s$), the irregular deposition is concentrated in a smaller area.

### 4.4. Effect of Nozzle Radius

Here we analyzed the effect of the nozzle radius, R, in the electrospinning process. The jet’s fiber radius increases as a function of the nozzle radius for R≥300 μm; as we can see in Figure 9a, the case R=200 μm bucks the trend with the largest fiber radius. In Figure 9b, we show the x and y coordinates where the jet hits the collector; these coordinates draw regular circles whose radius increase as R increases except for the R=200 μm. In this case, the deposition area is smaller. It seems that the external effects do not influence the electrospinning process with a small nozzle radius. We need to point out that this modeling approach is more appropriate for qualitative description than an exact reproduction of the experiments, and it does not include Taylor cone effects (i.e., the distortion of the droplet into a conical shape due to accumulation of charge). It means that in JETSPIN software, the initial jet cross-section at the nozzle $\left(a_{0}\right)\;$is an input parameter instead of following other models in equating it to the nozzle radius, R. Since R is usually larger than $a_{0}$, the JETSPIN software developers recommend setting $a_{0}=R/5,$ and we have adopted this empirical reduction value for $a_{0}$.

### 4.5. Effect of AC Electrospinning

In this study, we also considered the effect of AC electrospinning under four types of external electric fields: a static electric potential, a rectangular wave (Figure 10) oscillating with frequency $f_{s}$ oriented along the *z*-axis as expressed in Equation (9) in terms of $x\left(t\right)=\prod \left(\frac{t}{\tau }-\frac{1}{2}\right)$ that denotes a unit rectangular pulse starting at the origin with a width of $\tau =\frac{1}{2f_{s}}$ where $\prod \left(T\right)=\{\begin{array}{cc} 1 & \left|T\right|<\frac{1}{2}\\ 0 & \mathrm{otherwise} \end{array}$. The rectangular waveform is smoothed with rising and falling edges in Equation (10) to account for the resistance–capacitance (RC) relaxation of a cable in response to x(t) which is replaced by the relaxed response $y\left(t\right)=\left[1-e^{-\frac{t}{RC}}\;\right]x\left(t\right)+e^{-\frac{t}{RC}}\left[e^{\frac{\tau }{RC}}-1\right]x\left(t-\tau \right)$ (see Figure 9). Equation (11) includes a rotating electric field with the frequency $f_{s}$ on the transverse plane

(9) $$E=\left(0,0,E_{z}\overset{\infty }{\underset{n=0}{\sum }}x\left(t-\frac{n}{f_{s}}\right)\;\right)$$

(10) $$E=\left(0,0,E_{z}\overset{\infty }{\underset{n=0}{\sum }}y\left(t-\frac{n}{f_{s}}\right)\right)\;$$

(11) $$E=\left(E_{x}\sin\left(2\pi f_{s}t\right)\;,E_{y}\cos(2\pi f_{s}t)\;,E_{z}\right)$$

**Figure 11:**
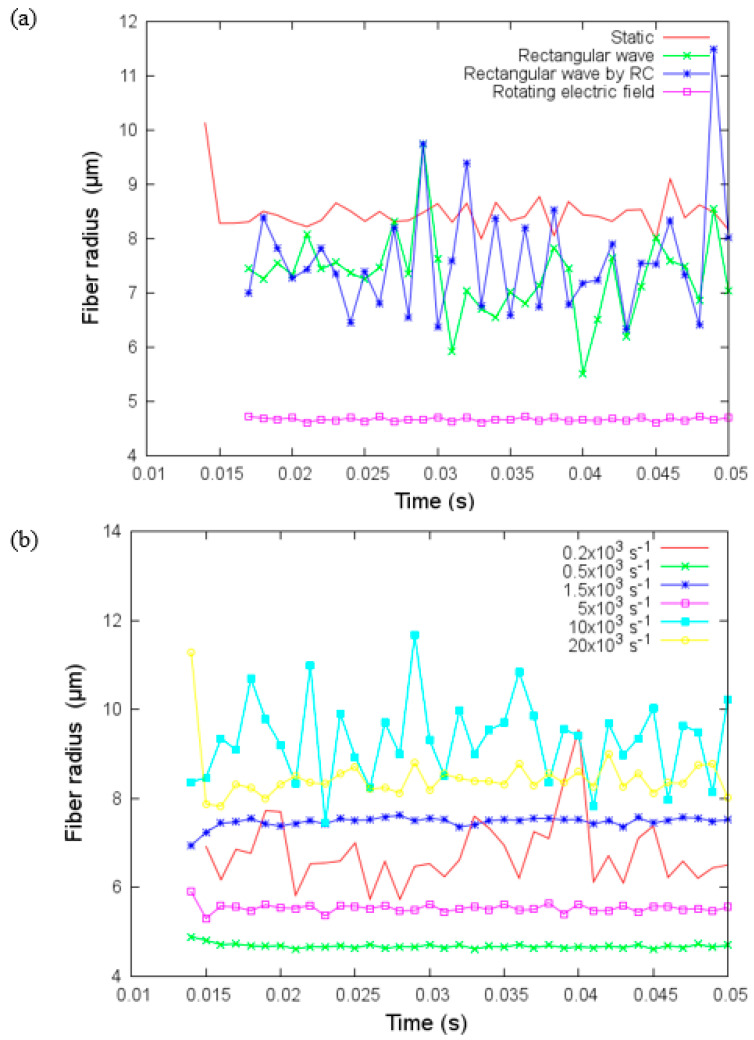
(**a**) Fiber radius versus time for four different electric fields with $f_{s}$ = $0.5\times 10^{3}\;\mathrm{Hz}$ and (**b**) Fiber radius versus time for different values of frequency $f_{s}$ for the rotating electric field on the transverse plane under parameters given in Table 3.

The effect of the rotating electric field has been demonstrated previously [32]. In Figure 11a, we show the fiber radius of the jet versus time for these four different electric fields with $f_{s}=0.5\times 10^{3}\;\mathrm{Hz},\;$relaxation time constant RC=0.2 ms, and $V_{x}=6\;\mathrm{kV},\;V_{y}=8\;\mathrm{kV}$, $V_{z}=9\;\mathrm{kV}$ and h = 10 cm. For rectangular wave fields, the fiber radius presents significant variations, but these variations are not present in the case of a rotating electric field. Moreover, the fiber radius is smallest when a rotating electric field is used, and the biggest radius is for the static case. Figure 11b shows the fiber radius against time for different values of frequency. There is no monotonic tendency for the variation of the fiber radius with the frequency $f_{s}$. For example, at $f_{s}=0.2\times 10^{3},\;10\times 10^{3}\;\mathrm{and}\;20\times 10^{3}$ Hz the fiber radius exhibits significant variations over the observation window that contrasts with the other three frequencies at which almost constant fiber radii can be obtained within a short period. The smallest fiber radius can be reached for the case $f_{s}=0.5\times 10^{3}\;\mathrm{Hz}$ while the largest fiber radius can be attained for the case $f_{s}=10\times 10^{3}\;\mathrm{Hz}$.

### 4.6. Correlation to Experimental Observations

The key parameters (such as distance at the collector, viscosity, the applied voltage, and needle diameter) identified by the simulation were adjusted to produce significant quantities of fiber using the 3 polymers examined, A85 (Huntsman Irogran A85–4350, Thermoplastic polyurethane), SBS (Sigma-Aldrich Polystyrene/polybutadiene, 30% polystyrene), SIS-17 (Sigma-Aldrich Polystyrene/polyisoprene, 17% polystyrene) and the two solvents, DMF (N,N-Dimethylformamide) and DCE (1,2-Dichloroethane). The simulations allowed us to optimize the jet spinning process to produce more desirable fiber features and reduced the experimentation required. Experimental observations confirmed the results obtained from the simulation on the impact of the voltage applied, needle diameter, and flow rate. The simulations from air drag also show how the air velocity can significantly impact the radius of the fiber and the location the fiber hits the collector plate. Stable parameters for electrospinning conductively filled polymers are a distance between needle and collector of 2.5 cm, pump flow rate vs. cross-section of 2–4 mL/h, spinning voltage of 5–10 kV, and linear collector feed rate of 400–600 mm/s. As seen in Figure 3, the spun fiber is very fine (around 2 μm) and often meshed/tangled together.

## 5. Conclusions

In this work, we have analyzed the electrospinning process in a variety of conditions and used the numerical analysis to guide the process for producing stable electrospun dielectric elastomer fibers. Experimental results were shown to illustrate the fibers produced. The main objective of this study was to investigate how the processing parameters may be modeled and how that may impact the electrospinning process. Subsequent work on the DE fiber characterization will require enough specimens to produce statistically significant results and will need to include not only the geometrical features of the fibers along different lengths, but also the mechanical and electrical properties which are critical to the success of the DE fibers. The simulations from a versatile open-source platform show that the jet’s fiber radius is highly dependent on the voltage, the distance at the collector, viscosity, and nozzle radius if the nozzle radius is larger than 300 μm. Moreover, when airflow velocity is added to the process, the fiber radius presents variations on time, and its size can be reduced. The oscillating electric field has a significant influence on the fiber radius. The electric fields generated by a rectangular wave result in fibers with notable variations in their radius but without a considerable change in their size when the electric field is static. In the case of a rotating electric field, the fiber radius might present variations on time for specific frequencies. For others, this might be almost constant and smaller compared with the static electric field and rectangular wave. These types of models are suitable for a qualitative description than an exact quantitative reproduction of the experiments. Stable parameters based on the simulations allowed for electrospinning conductively filled polymers with a consistent fiber diameter of approximately 2 μm. In simulations, we used an empirical reduction factor of 1/5 of the nozzle radius to set up the initial jet cross-section. In future studies, we should consider other reduction factors for the different polymer fluids, and this will require a systematic study using high speed imaging to capture the real effects at the nozzle. Such experiments can then be used to better understand the behavior of the fluids near the nozzle. The future studies should also focus on investigating the impact of solvent evaporation, molecular chain movement, and account for their effect in the simulation.

## Figures and Tables

**Table 1:** Viscosity measurements for unfilled SBS solution and filled multiwalled carbon nanotubes (MWCNT) loaded A85 solution, showing shear-thinning behavior for unfilled and shear-thickening behavior for filled solutions.

Shear Rate (mm/s)	SBS Viscosity (cps)	A85 + 30% MWCNT Viscosity (cps)
0.196	14080	4330
0.392	14080	5320
0.982	13620	5990
1.96	10740	7300
3.92	9960	9560
7.85	8960	16220
19.6	8680	16890

**Table 2:** Parameters used to electrospun of A85 + 10 wt.% Carbon black fiber (needle diameter: 2 mm).

Concentration of Solid in Solvent	Distance between Needle and Collector (cm)	Pump Flow Rate (mL/h)	Spinning Voltage (kV)	Diameter Range (µm)
30 wt.%	2.5	2	5	1.5–2.6
30 wt.%	2.5	2	10	1.3–2.9
30 wt.%	2.5	4	5	1.5–2.8
30 wt.%	2.5	8	10	1.3–2.9

**Table 3:** Baseline simulation parameters used in the electrospinning process.

Young’s modulus (G)	1.32 MPa	$\mathrm{Applied}\;\mathrm{voltage}\;V_{0}$	6 kV
Distance at the collector (h)	10 cm	Viscosity μ	2.0 Pa·s
Density mass ρ	1030 kg/m^3^	$\mathrm{Fiber}\;\mathrm{radius}\;\mathrm{at}\;\mathrm{the}\;\mathrm{nozzle},\;a_{0}$	40 microns
Nozzle radius, R	200 microns	Frequency perturbation f	10^4^ s^−1^
$\mathrm{Air}\;\mathrm{density}\;\rho_{a}$	1.21 kg/m^3^	Amplitude perturbation A	10^−3^
$\mathrm{Kinematic}\;\mathrm{viscosity}\;\nu_{a}$	0.151 cm^2^/s	Surface perturbation α	21.1 mN/m

## Data Availability

Data obtained is contained within the article.
